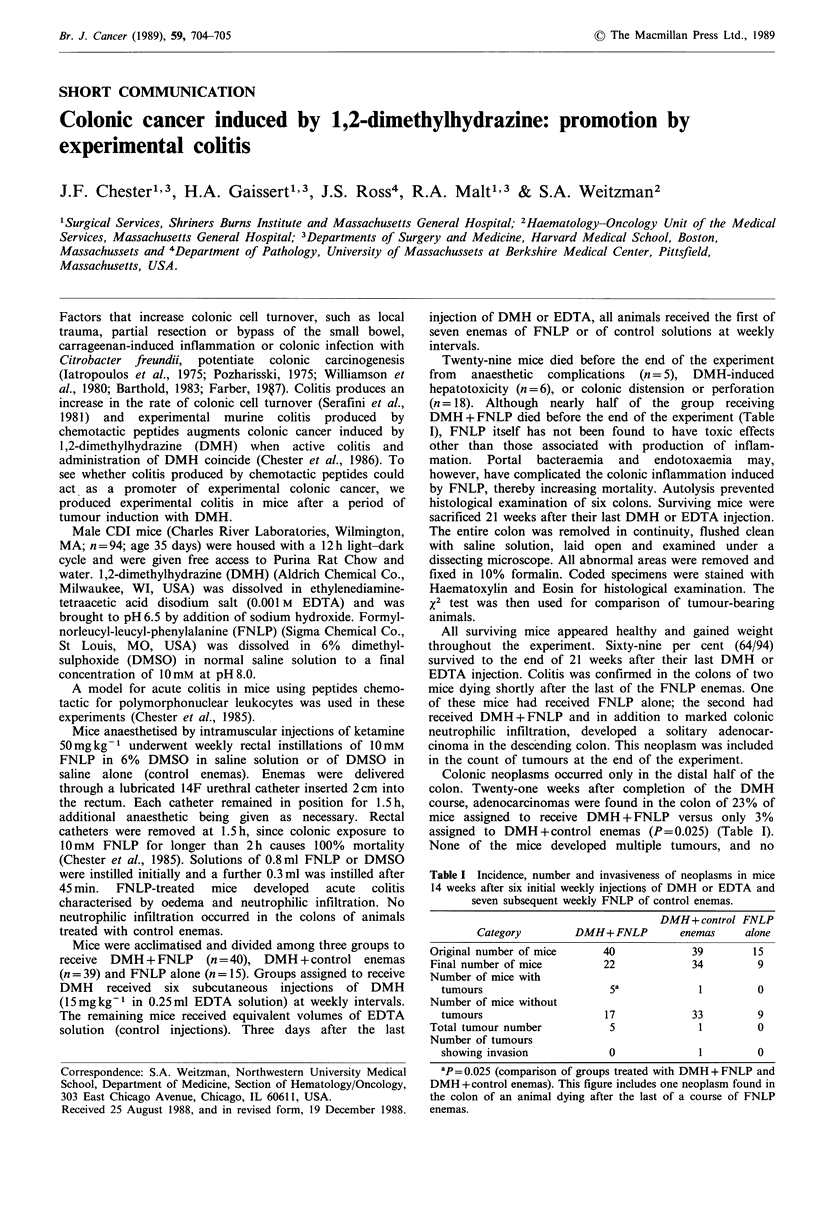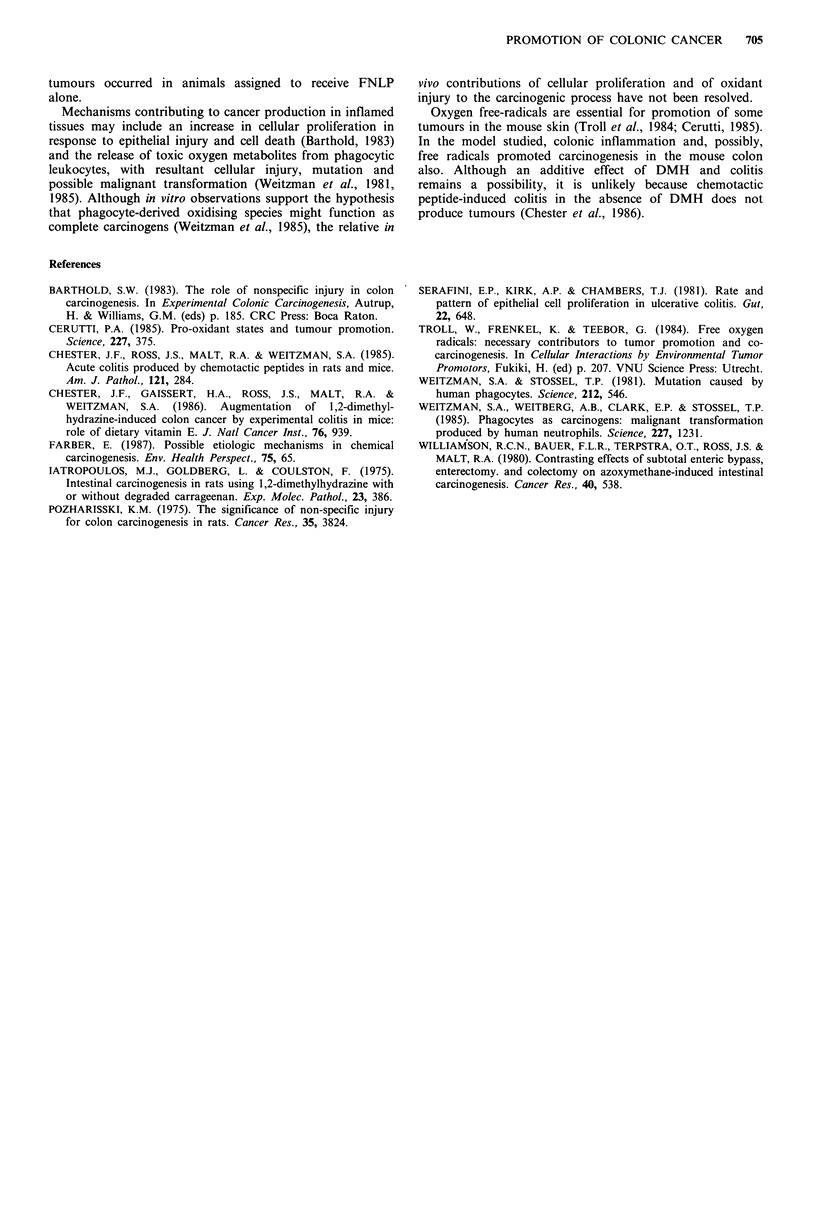# Colonic cancer induced by 1,2-dimethylhydrazine: promotion by experimental colitis.

**DOI:** 10.1038/bjc.1989.147

**Published:** 1989-05

**Authors:** J. F. Chester, H. A. Gaissert, J. S. Ross, R. A. Malt, S. A. Weitzman

**Affiliations:** Surgical Services, Shriners Burns Institute, Boston, Massachusetts.


*Br. J. Cancer* (1989), **59**, 704–705 © The Macmillan Press Ltd., 1989

SHORT COMMUNICATION

### Colonic cancer induced by 1,2-dimethylhydrazine: promotion by experimental colitis

J.F. Chester[1,3], H.A. Gaissert[1,3], J.S. Ross[4], R.A. Malt[1,3] & S.A. Weitzman[2]

[1]*Surgical Services, Shriners Burns Institute and Massachusetts General Hospital;* [2]*Haematology–Oncology Unit of the Medical Services, Massachusetts General Hospital;* [3]*Departments of Surgery and Medicine, Harvard Medical School, Boston, Massachussets and* [4]*Department of Pathology, University of Massachussets at Berkshire Medical Center, Pittsfield, Massachusetts, USA.*

Factors that increase colonic cell turnover, such as local trauma, partial resection or bypass of the small bowel, carrageenan-induced inflammation or colonic infection with *Citrobacter freundii*, potentiate colonic carcinogenesis (Iatropoulos *et al*., 1975; Pozharisski, 1975; Williamson *et al*., 1980; Barthold, 1983; Farber, 1987). Colitis produces an increase in the rate of colonic cell turnover (Serafini *et al*., 1981) and experimental murine colitis produced by chemotactic peptides augments colonic cancer induced by 1,2-dimethylhydrazine (DMH) when active colitis and administration of DMH coincide (Chester *et al*., 1986). To see whether colitis produced by chemotactic peptides could act as a promoter of experimental colonic cancer, we produced experimental colitis in mice after a period of tumour induction with DMH.

Male CDI mice (Charles River Laboratories, Wilmington, MA; $n=94$; age 35 days) were housed with a 12 h light–dark cycle and were given free access to Purina Rat Chow and water. 1,2-dimethylhydrazine (DMH) (Aldrich Chemical Co., Milwaukee, WI, USA) was dissolved in ethylenediamine-tetraacetic acid disodium salt (0.001 M EDTA) and was brought to pH 6.5 by addition of sodium hydroxide. Formyl-norleucyl-leucyl-phenylalanine (FNLP) (Sigma Chemical Co., St Louis, MO, USA) was dissolved in 6% dimethyl-sulphoxide (DMSO) in normal saline solution to a final concentration of 10 mM at pH 8.0.

A model for acute colitis in mice using peptides chemotactic for polymorphonuclear leukocytes was used in these experiments (Chester *et al*., 1985).

Mice anaesthetised by intramuscular injections of ketamine $50\,mg\,kg^{-1}$ underwent weekly rectal instillations of 10 mM FNLP in 6% DMSO in saline solution or of DMSO in saline alone (control enemas). Enemas were delivered through a lubricated 14F urethral catheter inserted 2 cm into the rectum. Each catheter remained in position for 1.5 h, additional anaesthetic being given as necessary. Rectal catheters were removed at 1.5 h, since colonic exposure to 10 mM FNLP for longer than 2 h causes 100% mortality (Chester *et al*., 1985). Solutions of 0.8 ml FNLP or DMSO were instilled initially and a further 0.3 ml was instilled after 45 min. FNLP-treated mice developed acute colitis characterised by oedema and neutrophilic infiltration. No neutrophilic infiltration occurred in the colons of animals treated with control enemas.

Mice were acclimatised and divided among three groups to receive DMH + FNLP ($n=40$), DMH + control enemas ($n=39$) and FNLP alone ($n=15$). Groups assigned to receive DMH received six subcutaneous injections of DMH ($15\,mg\,kg^{-1}$ in 0.25 ml EDTA solution) at weekly intervals. The remaining mice received equivalent volumes of EDTA solution (control injections). Three days after the last injection of DMH or EDTA, all animals received the first of seven enemas of FNLP or of control solutions at weekly intervals.

Twenty-nine mice died before the end of the experiment from anaesthetic complications ($n=5$), DMH-induced hepatotoxicity ($n=6$), or colonic distension or perforation ($n=18$). Although nearly half of the group receiving DMH + FNLP died before the end of the experiment (Table I), FNLP itself has not been found to have toxic effects other than those associated with production of inflammation. Portal bacteraemia and endotoxaemia may, however, have complicated the colonic inflammation induced by FNLP, thereby increasing mortality. Autolysis prevented histological examination of six colons. Surviving mice were sacrificed 21 weeks after their last DMH or EDTA injection. The entire colon was removed in continuity, flushed clean with saline solution, laid open and examined under a dissecting microscope. All abnormal areas were removed and fixed in 10% formalin. Coded specimens were stained with Haematoxylin and Eosin for histological examination. The $\chi^2$ test was then used for comparison of tumour-bearing animals.

All surviving mice appeared healthy and gained weight throughout the experiment. Sixty-nine per cent (64/94) survived to the end of 21 weeks after their last DMH or EDTA injection. Colitis was confirmed in the colons of two mice dying shortly after the last of the FNLP enemas. One of these mice had received FNLP alone; the second had received DMH + FNLP and in addition to marked colonic neutrophilic infiltration, developed a solitary adenocarcinoma in the descending colon. This neoplasm was included in the count of tumours at the end of the experiment.

Colonic neoplasms occurred only in the distal half of the colon. Twenty-one weeks after completion of the DMH course, adenocarcinomas were found in the colon of 23% of mice assigned to receive DMH + FNLP versus only 3% assigned to DMH + control enemas ($P=0.025$) (Table I). None of the mice developed multiple tumours, and no

**Table I** Incidence, number and invasiveness of neoplasms in mice 14 weeks after six initial weekly injections of DMH or EDTA and seven subsequent weekly FNLP of control enemas.

| *Category* | *DMH + FNLP* | *DMH + control enemas* | *FNLP alone* |
|---|---|---|---|
| Original number of mice | 40 | 39 | 15 |
| Final number of mice | 22 | 34 | 9 |
| Number of mice with tumours | 5[a] | 1 | 0 |
| Number of mice without tumours | 17 | 33 | 9 |
| Total tumour number | 5 | 1 | 0 |
| Number of tumours showing invasion | 0 | 1 | 0 |

[a]$P=0.025$ (comparison of groups treated with DMH + FNLP and DMH + control enemas). This figure includes one neoplasm found in the colon of an animal dying after the last of a course of FNLP enemas.

Correspondence: S.A. Weitzman, Northwestern University Medical School, Department of Medicine, Section of Hematology/Oncology, 303 East Chicago Avenue, Chicago, IL 60611, USA.
Received 25 August 1988, and in revised form, 19 December 1988.

tumours occurred in animals assigned to receive FNLP alone.

Mechanisms contributing to cancer production in inflamed tissues may include an increase in cellular proliferation in response to epithelial injury and cell death (Barthold, 1983) and the release of toxic oxygen metabolites from phagocytic leukocytes, with resultant cellular injury, mutation and possible malignant transformation (Weitzman *et al.*, 1981, 1985). Although *in vitro* observations support the hypothesis that phagocyte-derived oxidising species might function as complete carcinogens (Weitzman *et al.*, 1985), the relative *in vivo* contributions of cellular proliferation and of oxidant injury to the carcinogenic process have not been resolved.

Oxygen free-radicals are essential for promotion of some tumours in the mouse skin (Troll *et al.*, 1984; Cerutti, 1985). In the model studied, colonic inflammation and, possibly, free radicals promoted carcinogenesis in the mouse colon also. Although an additive effect of DMH and colitis remains a possibility, it is unlikely because chemotactic peptide-induced colitis in the absence of DMH does not produce tumours (Chester *et al.*, 1986).

### References

BARTHOLD, S.W. (1983). The role of nonspecific injury in colon carcinogenesis. In *Experimental Colonic Carcinogenesis*, Autrup, H. & Williams, G.M. (eds) p. 185. CRC Press: Boca Raton.

CERUTTI, P.A. (1985). Pro-oxidant states and tumour promotion. *Science*, **227**, 375.

CHESTER, J.F., ROSS, J.S., MALT, R.A. & WEITZMAN, S.A. (1985). Acute colitis produced by chemotactic peptides in rats and mice. *Am. J. Pathol.*, **121**, 284.

CHESTER, J.F., GAISSERT, H.A., ROSS, J.S., MALT, R.A. & WEITZMAN, S.A. (1986). Augmentation of 1,2-dimethylhydrazine-induced colon cancer by experimental colitis in mice: role of dietary vitamin E. *J. Natl Cancer Inst.*, **76**, 939.

FARBER, E. (1987). Possible etiologic mechanisms in chemical carcinogenesis. *Env. Health Perspect.*, **75**, 65.

IATROPOULOS, M.J., GOLDBERG, L. & COULSTON, F. (1975). Intestinal carcinogenesis in rats using 1,2-dimethylhydrazine with or without degraded carrageenan. *Exp. Molec. Pathol.*, **23**, 386.

POZHARISSKI, K.M. (1975). The significance of non-specific injury for colon carcinogenesis in rats. *Cancer Res.*, **35**, 3824.

SERAFINI, E.P., KIRK, A.P. & CHAMBERS, T.J. (1981). Rate and pattern of epithelial cell proliferation in ulcerative colitis. *Gut*, **22**, 648.

TROLL, W., FRENKEL, K. & TEEBOR, G. (1984). Free oxygen radicals: necessary contributors to tumor promotion and co-carcinogenesis. In *Cellular Interactions by Environmental Tumor Promotors*, Fukiki, H. (ed) p. 207. VNU Science Press: Utrecht.

WEITZMAN, S.A. & STOSSEL, T.P. (1981). Mutation caused by human phagocytes. *Science*, **212**, 546.

WEITZMAN, S.A., WEITBERG, A.B., CLARK, E.P. & STOSSEL, T.P. (1985). Phagocytes as carcinogens: malignant transformation produced by human neutrophils. *Science*, **227**, 1231.

WILLIAMSON, R.C.N., BAUER, F.L.R., TERPSTRA, O.T., ROSS, J.S. & MALT, R.A. (1980). Contrasting effects of subtotal enteric bypass, enterectomy. and colectomy on azoxymethane-induced intestinal carcinogenesis. *Cancer Res.*, **40**, 538.